# Investigating the effect of a school-based WASH intervention on soil-transmitted helminth and schistosome infections and nutritional status of school children in Ethiopia: a quasi-experimental study

**DOI:** 10.1186/s13071-024-06155-2

**Published:** 2024-03-14

**Authors:** Gemechu Tadesse, Yonas Wuletaw, Kalkidan Mekete, Heven Sime, Elodie Yard, Laura Appleby, Jack Grimes, Nigussie Dejene, Iain Gardiner, Adama Kazienga, Souheila Abbeddou, Michael French, Bruno Levecke, Lesley Drake

**Affiliations:** 1 Ethiopian Public Health Institute, Addis Ababa, Ethiopia; 2 Department of Translational Physiology, Infectiology and Public Health, Faculty of Veterinary Medicine, Ghent University, Merelbeke, Belgium; 3 Partnership for Child Development, London, UK; 4 Department of Public Health and Primary Care, Faculty of Medicine and Health Sciences, Ghent University, Ghent, Belgium; 5 RTI International, Washington DC, USA; 6 Department of Civil and Environmental Engineering, South Kensington Campus, Imperial College London, London, UK

**Keywords:** Soil-transmitted helminths, Schistosomes, Deworming, WASH, Health education, Quasi-random experiment, Ethiopia

## Abstract

**Background:**

The impact of access to improved water, sanitation and hygiene (WASH) and health education on large-scale deworming programs aimed at controlling soil-transmitted helminth (STH) and schistosome (SCH) infections has not been well studied. We assessed the additional impact of improved WASH infrastructure and health education at schools on STH and SCH infections in Ethiopia.

**Methods:**

The study used a quasi-experimental design under which 30 schools were assigned to either an intervention (15 schools) or control (15 schools) arm. Both arms received a standard deworming treatment and lunch. In the intervention arm, improved WASH and health education were provided. At three consecutive time points (baseline in 2013, 2014 and 2015), the prevalence and intensity of STH and SCH infections and the nutritional status [hemoglobin concentrations and physical growth (height and weight)] were determined. To verify whether interventions were successfully implemented, the WASH status at school and the student knowledge, attitudes and practices related to WASH (WASH-KAP) were recorded. Differences in metrics between arms at baseline (2013) and follow-up (2015) were assessed both within and between the arms.

**Results:**

A significant increase in scores for both the school WASH and student KAP was found in the intervention arm, indicating successful implementation of the intervention. The prevalence of any STH infection was significantly reduced in the intervention arm but not in the control arm (F = 4.486, *p* = 0.034). There was a significantly greater reduction in the intensity of infection of hookworm and *Ascaris lumbricoides* compared to baseline in both arms. The intervention did not affect school children’s height-for-age z-score (intervention arm * time coef = 0.12, *p* = 0.400) and body mass index-for-age z-scores (intervention * time coef = − 0.06, *p* = 0.526). Hemoglobin concentrations increased significantly more in the control than the intervention arm (coef = − 0.16, *p* = 0.006).

**Conclusions:**

Although the intervention did increase school WASH and student WASH-KAP, our study found poor evidence of the additional benefit of improved WASH and health education to deworming and school food programs on parasite re-infection and the health outcomes of children.

**Graphical Abstract:**

**Figure:**
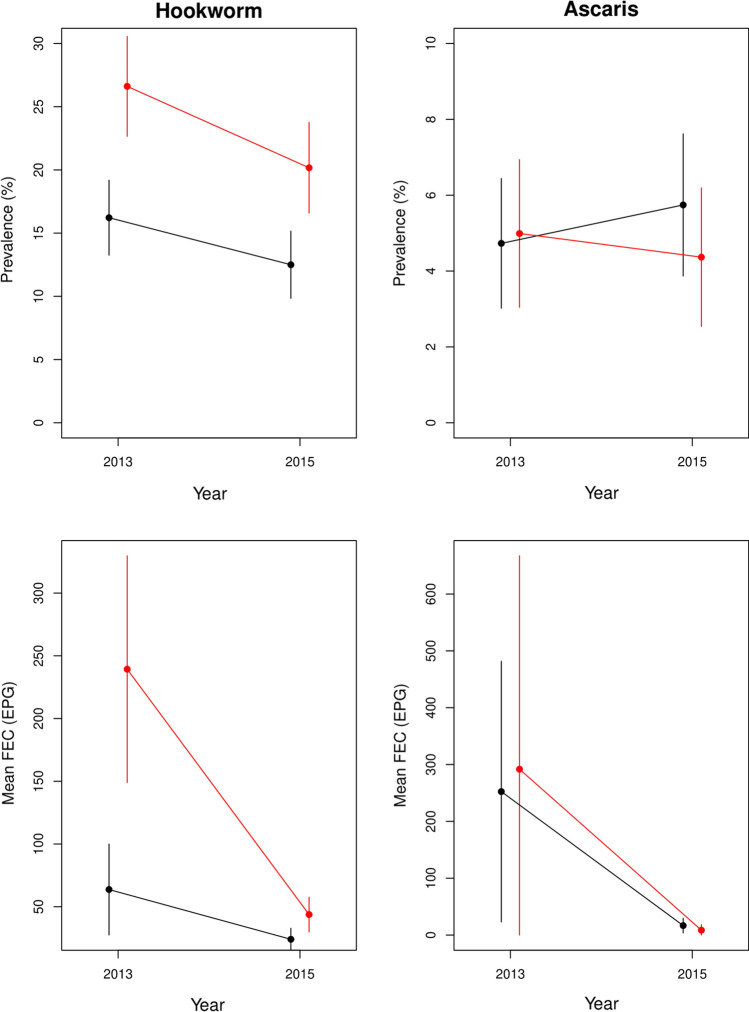

**Supplementary Information:**

The online version contains supplementary material available at 10.1186/s13071-024-06155-2.

## Background

Soil-transmitted helminthiases and schistosomiasis place significant burdens on public health. Soil-transmitted helminthiases are caused by a group of intestinal worms, *Ascaris lumbricoides*, *Trichuris trichiura* and two hookworm species (*Necator americanus* and *Ancylostoma duodenale*), whereas schistosomiasis is caused by the blood-dwelling worm *Schistosoma mansoni* (intestinal schistosomiasis) and *S. haematobium* (urogenital schistosomiasis). It is estimated that 1.6 billion and 252.3 million people worldwide suffer from infections with soil-transmitted helminths (STHs) and schistosomes (SCHs), resulting in a cumulative disease burden of 6.1 million disability-adjusted life-years (soil-transmitted helminthiases: 3.5 million; schistosomiasis: 2.6 million [1, 2]).

Given the nature of transmission (STHs: feco-oral/per cutaneous; SCHs: contact with water infested with the snail intermediate host), both diseases are common in communities that have limited access to clean water, sanitation and hygiene (WASH). Globally, 2.1 billion people lack safe drinking water. Indeed, 263 million people daily spend more than 30 min to collect water, and 159 million people drink untreated surface water from lakes, rivers or streams [3]. About 2.3 billion people have no basic sanitation services, 892 million people defecate in the open, and 600 million share toilets with other households [3]. In low-income countries, approximately half of the population has no access to handwashing facilities [3].

Four major sequelae are attributed to STH and SCH infections: abdominopelvic problems, symptomatic infection, wasting/thinness and anemia [4], with school children the most vulnerable [5]. The optimal strategy to control the transmission of both diseases is based on three pillars, (i) large-scale deworming to gradually reduce the infection burden, (ii) improved WASH to reduce environmental contamination with infectious stages and (iii) health education to alter behavior to reduce environmental contamination and risk of infection. Of the three, large-scale deworming programs have been the most widely implemented to date. In 2019, the World Health Organization (WHO) estimated that these programs treated 63% and 43%, respectively, of school children at risk of STH and SCH-attributable morbidity by periodically administering anthelmintic drugs (STHs: albendazole or mebendazole; SCHs: praziquantel) [6].

It has previously been documented that regular deworming reduces the prevalence and intensity of STH and SCH infections [7, 8]. The evidence regarding the additional health and nutritional benefits from improved WASH interventions however is mixed. An analysis of the Demographic and Health Survey (DHS) data from 65 countries estimated that a 20 percentage point reduction in open defecation was associated with a 0.1 standard deviation (SD) increase in the height-for-age z-score (HAZ) of children under five years [5]. Another systematic review reported a small benefit of WASH interventions on linear growth in children < 5 years of age [9]. Three more recent large-scale community randomized efficacy trials that tested the impact of household-level WASH with and without nutritional supplementation of mothers on child growth and diarrhea in Bangladesh [10], Kenya [11] and Zimbabwe [12] for 2 years did not report a significant benefit of WASH on the linear growth of children. Moreover, a rural sanitation program had no effect on mean HAZ in children < 2 years old at baseline in Odisha, India [13]. A review on the importance of WASH intervention and hygiene education on health outcomes of school children was inconclusive because of the limitations of the available studies [14]. A cross-sectional study conducted in the Philippines suggested that school children who were both stunted and undernourished had greater STH infections [15].

Although a reduction in disease transmission is expected for both diseases through these large-scale deworming programs, the additional impact of WASH and health education [16–19] on helminth infections and nutritional status remains unclear. Therefore, the present study aimed to investigate the impact of both WASH and health education interventions alongside school-based deworming and food programs primarily on (i) the prevalence and intensity of STH and SCH infections and secondarily on (ii) indicators of nutritional status [hemoglobin (Hb) concentrations, anemia, body mass index-for-age z-score (BMIAZ) and thinness, and HAZ and stunting] and (iii) improvements in the participants’ knowledge, attitudes and practices (KAP) of WASH to prevent STH and SCH infections.

## Methods

### Study area and design

A quasi-experimental study was conducted in rural areas of Segen, Wolaita, Gurage and Silte zones of the former Southern Nation, Nationalities Peoples Region (SNNPR), in Ethiopia and involved students aged 5–19 years who were enrolled from grade 1 to 6 at 30 government elementary schools. Elementary schools are often used for the implementation and evaluation of deworming programs as they offer a number of advantages: (i) the STH/SCH infection levels tend to peak in primary school children, (ii) the high enrollment rate in schools ensures that the majority of school children of the desired age group will be included in the sampling frame, minimizing selection bias, and (iii) schools present a convenient logistical platform for conducting surveys and delivering treatment. The schools included in this study were enrolled in a pilot project to test the feasibility of feeding school children through food that was produced locally by small land holders and therefore aimed to create opportunities to the farmers. The farmers were provided with improved seeds and fertilizers as an incentive through the Regional Office of the Food and Agriculture Organization (FAO) of the United Nations. These pilot schools are located in five zones of SNNPR (Segen: 11 schools, Wolaita: 4 schools, Gurage: 10 schools and Silte: 5 schools). The costs for food were financed by the United Nations World Food Program (UN-WFP) and coordinated by the Federal Ministry of Education and Regional Education Bureaus.

**Fig. 1:**
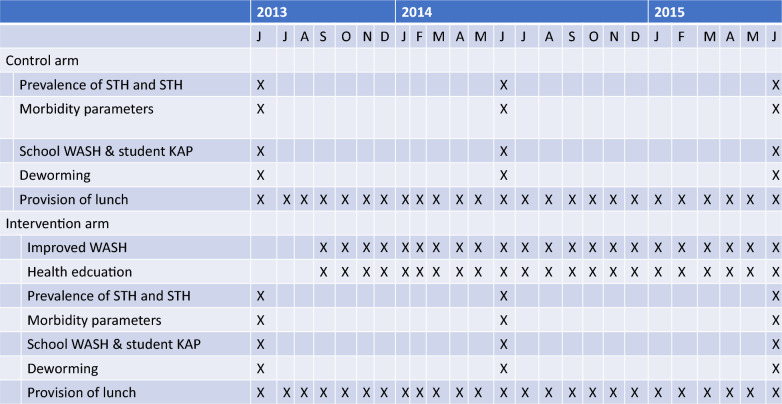
Schematic overview of the study activities

The schools were assigned to either the intervention (15 schools; Segen: 4 schools; Wolaita: 3 schools; Gurage: 5 schools and Silte: 3 schools) or the control arm (15 schools; Segen: 7 schools; Wolaita: 1 school; Gurage: 5 schools and Silte: 2 schools). The assignment of the schools to these arms was based solely on the availability of budget to upgrade WASH infrastructures for the intervention arm prior to the commencement of the study. During the 9-month school year, the intervention arm received a once-yearly administration of an anthelmintic drug (STHs: 400 mg of albendazole), improved WASH (provision of clean water by building water tanks to capture rain water, installing piped water and constructing latrines), lunch and behavior change education. The control arm received a once-yearly administration of an anthelmintic drug (STHs: 400 mg of albendazole) and lunch only. In both arms, schistosomiasis (*S. mansoni*)-positive cases received 40 mg praziquantel/kg body weight. Figure 1 provides an overview of the activities during the study (June 2013 to June 2015).

At the time of the baseline survey (June 2013), we determined (i) the prevalence and intensity of STH and SCH infections, (ii) nutritional indicators (Hb concentration, height and weight), (iii) school WASH metrics (e.g. availability of latrines, hand washing facilities and source of drinking water/school WASH) and (iv) the students’ KAP of WASH to prevent STH and SCH infections (student WASH-KAP). For both the infections and nutritional parameters, we randomly selected 125 school children per school who had their height and weight measured. This sample size of 125 school children per school was taken from the experience of monitoring the impact of deworming programs in sentinel sites after rounds of deworming as stated in the WHO guidelines to monitor and evaluate control programs [20]. Height was measured once to the nearest 0.1 cm using a portable height scale, and weight was assessed once to within 50 g using an electronic balance. In addition, stool and capillary blood samples were collected. Stool samples were screened for the presence and the number of STH and *S. mansoni* eggs (stool samples), while blood samples were used to determine the Hb concentration (Hb in g/dl). The school WASH components were assessed yearly at each school. For this, the survey team interviewed the school directors while completing a questionnaire. The team also made observations on the presence and status of the different WASH infrastructure components at the school. Students’ KAP towards WASH was assessed through administration of a questionnaire at each school to 15 of the 125 selected school children at all the survey points.

After the completion of the baseline survey in June 2013, a single oral dose of 400 mg albendazole for STH infections was provided to all students regardless of their infection status and 40 mg/kg of body weight of praziquantel to school children infected with *S. mansoni* [21]. Before administering the drugs, the school children were briefed on the benefits of the drugs, and time was given for the school children to ask any questions about the drugs to avoid any ambiguity and misinformation. The administration of drugs was implemented by the survey health officers in collaboration with the Woreda Health Office and school director, as at that time there was not yet a nationwide control program for STHs and SCHs [22]. In the follow-up surveys (June 2014 and June 2015), the same participants were followed up, and the prevalence and intensity of STH and SCH infections, nutritional indicators, school WASH and school children’s KAP towards WASH were re-assessed.

Throughout the study, a number of intervention activities were applied, including (i) annually deworming school children at all schools (June 2014 and June 2015), (ii) providing a lunch (porridge made of milled maize, boiled maize and haricot bean mix, and kinche/cracked wheat; this was part of the UN-WFP supported program) on all school days (Monday to Friday) during the school year and (iii) improving the school WASH facilities in the intervention schools. For the latter, latrines and handwashing facilities were constructed, and clean water provision was ensured by, for example, installing new water pipelines. All the WASH interventions were implemented by the Dutch non-profit development organization ‘Stichting Nederlandse Vrijwilligers’ (SNV; Foundation of Netherlands Volunteers), with financial support from Dubai Cares International. SNV also provided education tools (posters, flyers, mini media platforms, plays and songs) to improve personal hygiene behaviors and reduce environmental contamination.

### Operational procedures

#### Assessment of prevalence and intensity of soil-transmitted helminths and *S. mansoni* infections

Stool samples from 125 students in each of the 30 schools were examined applying a single Kato-Katz thick smear (41.7 mg template) to assess the number of *Ascaris*, *Trichuris*, hookworms and *S. mansoni* eggs [23]. The number of eggs was multiplied by 24 to obtain the fecal egg counts [FECs; expressed as eggs per gram of stool (EPG)] [24]. As part of quality control, 10% of the Kato-Katz thick smears were randomly selected and re-examined by a senior laboratory technician, and timely feedback was provided to the survey teams in case of discrepancies. Note that *S. haematobium* is not present in this area; hence, we did not perform any laboratory tests to assess its prevalence or intensity.

#### Assessment of nutritional indicators

Capillary blood samples (10 μl) were collected from a finger prick with a sterile disposable lancet (Haemolance Plus; 1.8-mm puncture depth). Hb concentration was immediately analyzed using the point-of-care HemoCue (HemoCueAB, Ängelholm, Sweden). Subjects were classified as non-anemic, mildly, moderately or severely anemic applying the standardized age- and sex-adjusted WHO cutoffs [25]. These were adjusted for altitude using the following method: Hb adjustment = 0.32 × (altitude in meters × 0.0033) + 0.22 × (altitude in meters × 0.0033)^2^ [26]. This value was subtracted from the raw Hb value to provide a corresponding value at sea level.

The quality of anthropometric data was checked and assessed by verifying whether changes in height and age were plausible across the different surveys. We only included those subjects for whom the recorded sex remained unchanged, the age increased by 12 months and the recorded height at the follow-up surveys did not drop > 1.0 cm below the height recorded at baseline, and the difference in height between respective surveys was ≥ 1.0 cm. The nutritional status of the subjects was calculated based on age, sex, height and weight. Both HAZ and BMIAZ were calculated in relation to the WHO Child Growth Standards using R macros to calculate the nutritional status of children using age, sex, height and weight based on the WHO Child Growth Standard for children aged 5–19 years [27]. Moderate stunting and thinness were defined as 2 SD below the mean HAZ and BMIAZ, respectively, and severe stunting and thinness were defined as 3 SD below the mean [27].

#### Assessment of school WASH and student WASH-KAP

We assessed the WASH status of the schools and KAP of students towards WASH through questionnaires. The questionnaires are provided in the Additional file 1 and Additional file 2. For the assessment of WASH, we developed two separate questionnaires. The first asked about the availability and use of water, latrines and handwashing facilities at schools (school WASH). The second, the student WASH-KAP questionnaire, obtained student information from the 15 randomly selected students from each school. The student WASH-KAP questionnaire consisted of multiple-choice and open-ended questions designed to elicit insights into students’ KAP towards WASH. The topics were personal hygiene practices (e.g. hand washing, food handling, toilet use and open defecation practice) and access to water. The age range of the students included in this WASH-KAP questionnaires was 7 to 18 (mean ± SD: 12.0 ± 1.9) years. For both the school WASH and WASH-KAP questionnaires, we determined a total score based on the composite of the binary responses to each individual question, with higher scores indicating a stronger WASH infrastructure or improved student KAP towards WASH. Each question scored either 1 or 0, and the total maximum score was 17 for the school WASH questionnaire and 14 for the student WASH-KAP questionnaire.

#### Data collection and management

All data were captured using paper record forms. Double data entry was completed using CS-Pro version 5 (United States Census Bureau, Washington, DC, USA). Subsequently, databases were exported to SPSS version 16 (IBM Corp., Armonk, NY) for further data curation. Following completion of the data entry, the two databases were reconciled, and any discrepancies were investigated and resolved by referring to the original forms. Additionally, during data collection a team of independent experts undertook the surveys simultaneously with a random data collector team, moving around each team over the period of data collection. This allowed the data collection accuracy to be compared on site, with discrepancies being discussed and corrected when required. The final statistical data analyses were conducted in R version 3.5.1 (R Foundation for Statistical Computing, Vienna, Austria) [28].

### Statistical data analysis

The statistical analysis was conducted in four steps. First, we summarized the demographic characteristics (age and sex), prevalence and intensity of STH and SCH infections, nutritional indicators (Hb concentration and anemia, HAZ and stunting, and BMIAZ and thinness) and both the school WASH and student WASH-KAP at baseline for each of the two arms separately. For continuous variables (age, intensity of STH and SCH infections, Hb concentration, HAZ, BMIZ, WASH and the student WASH-KAP), we reported means (± SD) or median (in case data were not normally distributed based on the output of the Shapiro-Wilk test and Q-Q plot). For categorical variables (prevalence of STH and SCH infections, anemia, stunting and thinness), we reported proportions. Then, a univariate analysis was conducted to explore baseline differences in these variables between the two arms. To this end, we used Student’s t-test (normally distributed data: Hb concentration) and Mann-Whitney U test (non-normally distributed data: age, HAZ, BMIAZ) for continuous variables and chi-square test for categorical data.

Second, we verified whether the WASH and student WASH-KAP score improved in the intervention arm over time to assess whether WASH and education interventions were successfully implemented. For this, we applied a Wilcoxon signed rank test to verify any difference in school WASH and WASH-KAP between baseline (2013) and endline (2015). We also explored spillover effect using differences in the difference between the intervention and control arms applying a linear mixed model.

Third, we assessed the impact of the intervention on the occurrence of any STH infections and indicators of nutritional status (HAZ, BMIAZ and Hb concentration). Due to the low prevalence of *S. mansoni*, this analysis was restricted to STH infections only. For the occurrence of helminth infections, we used binomial generalized linear mixed model accounting for the school effect and the repeated measures made on the same children, where the occurrence of any STH infection (present or absent) represented the dependent variable, and age at baseline (in years), the arm (intervention vs. control), baseline WASH score, change in WASH score, sex (male vs. female), time (2013, 2014 and 2015) and an interaction term between the time and arms as potential predictors. For the variables reflecting nutritional status, we used linear mixed models accounting for the school level effect and the repeated measures made on the same children, where the nutritional status variable represented the dependent variable, and age at baseline (in years), arm (intervention vs. control), baseline school WASH score, change in WASH score, sex (male vs. female), any STH infection (present vs. absent), time (2013, 2014 and 2015) and an interaction term between the time and intervention as the potential predictors. Separate models were built for each variable reflecting nutritional status.

Finally, we summarized the demographic characteristics (age and sex), prevalence and mean FECs (in EPG) of STH and SCH infections, nutritional indicators (Hb concentration and anemia, HAZ and stunting, and BMIAZ and thinness) and both the school WASH and student WASH-KAP at baseline for school children that completed the study and those that were lost to follow-up. Then, a univariate analysis was conducted to explore baseline differences in these variables between the datasets as described above.

## Results

### Characteristics of the school children and schools at baseline

**Fig. 2:**
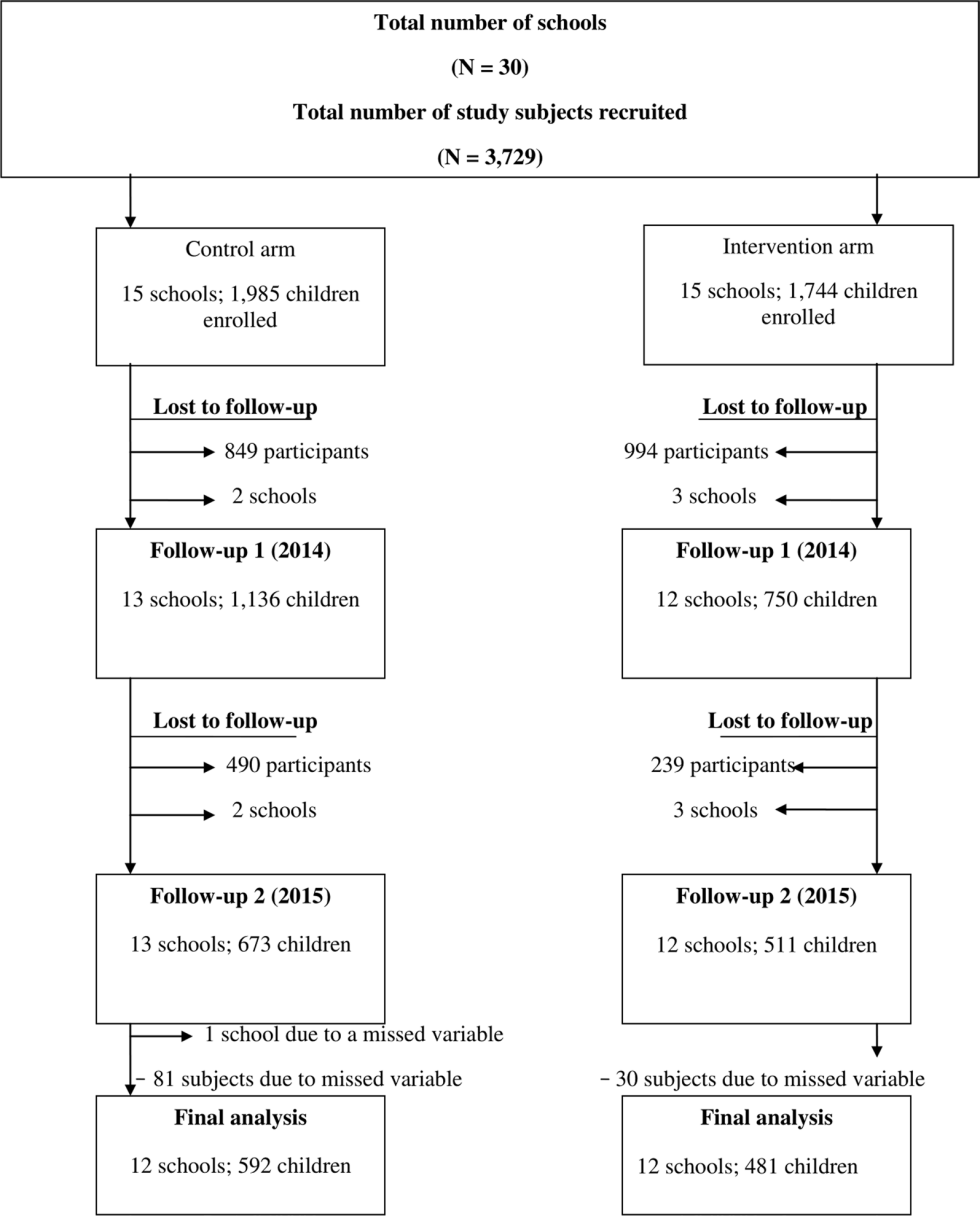
The number of school children recruited, lost to follow-up and incorporated into the data analysis

Figure 2 provides an overview of the number of school children who were recruited at baseline, those lost to follow-up and those incorporated into the final data analysis. A total of 3729 school children were recruited from 30 schools, of which 1955 were in the control arm (15 schools) and 1774 were in the intervention arm. In total, 2229 (59.8%) school children were lost to follow-up [follow-up survey 1 (2014): 1843 across 25 schools; follow-up survey 2 (2015): 386 across 25 schools]. This high dropout rate was mainly due to the safety concerns for the field teams during the follow-up surveys. In addition, data collected from 374 (10.0%) school children were dropped from the final data set as their records were incomplete or they were > 19 years old, resulting in 1073 complete cases for the assessment of prevalence and intensity of helminth infections, and Hb concentration (592 in the control arm and 481 in the intervention arm), 24 complete schools (12 in the control arm and 12 in the intervention arm) for the school WASH questionnaire and 209 (of the 596 recruited subjects) for the student WASH-KAP questionnaire. Measures for both weight and height were available for only 567 subjects; for the other 506 students, either the data were missing or they did not pass the quality control.

The demographic characteristics (age and sex), prevalence and intensity of STH and SCH infections, and nutritional indicators at baseline are summarized in Table 1. Overall, 56.8% of the 1073 school children who completed the study were boys, and the mean age (range) of the school children was 11.7 (7–19) years. We observed any STH infection in 25.6% of the school children, hookworm infections being the most prevalent (20.9%), followed by *A. lumbricoides* (4.8%). Infections with *T. trichiura* (0.8%) were the least prevalent among STH species. *Schistosoma mansoni* infections were only observed in a few cases (0.2%). Moderate-to-heavy STH infections were found in 31 school children (2.9%) [20]. All *S. mansoni* infections were of low intensity. At baseline, 57.0% of the school children were anemic, including 2.4% who were severely anemic and 31.7% who were moderately anemic. A total of 16.9% and 11.2% of the school children had stunted growth (HAZ < –2 SD) or thin (BMIAZ < −2 SD), respectively.

**Table 1:** The demographic characteristics, helminth infections and nutritional status across the complete cases at baseline

	Metric	Value	Total (n = 1073)	Control arm (n = 592)	Intervention arm (n = 481)	*p*-value for difference between arms
*Demography*						
	Male (%)		56.8	55.9	57.8	0.536
	Mean age [SD]		11.7 [1.9]	11.7 [2.0]	11.7 [1.8]	0.873
*Helminth infection*						
	Any STH	Prevalence (%)	25.6	21.6	30.6	**0.001**
	*Ascaris*	Prevalence (%)	4.8	4.7	5.0	0.844
		Mean FEC (EPG)	270.1	252.4	291.8	0.861
	*Trichuris*	Prevalence (%)	0.7	1.4	0.0	**0.005**
		Mean FEC (EPG)	7.0	12.7	0	0.072
	Hookworm	Prevalence (%)	20.9	16.2	26.6	**0.000**
		Mean FEC (EPG)	142.4	63.7	239.3	**0.000**
	*Schistosoma mansoni*	Prevalence (%)	0.2	0.3%	0.0%	0.158
		Mean FEC (EPG)	< 0.1	0.08	0	0.158
*Nutritional indicators*						
	Hemoglobin concentration	g/dl [SD]	11.4 [1.5]	11.4 [1.4]	11.3 [1.6]	0.058
	Anemia	Severe (%)	2.4	1.5	3.5	**0.041**
		Moderate (%)	31.7	31.9	31.4	0.852
		Mild (%)	22.9	23.1	22.7	0.852
	BMIAZ	Mean [SD]	-0.74 [1.08]	-0.81 [1.03]	-0.65 [1.14]	**0.019**
	Thinness	Severe (< -3 SD)	1.7	1.5	1.9	0.660
		Moderate ([ -2 SD, -3 SD])	9.5	10.3	8.5	0.319
	HAZ	Mean [SD]	-0.66 [1.51]	-0.66 [1.54]	-0.65 [1.48]	0.948
	Stunting	Severe (< -3 SD)	5.9	6.6	5.0	0.262
		Moderate ([ -2 SD, -3 SD])	11.0	11.8	10.0	0.333

FEC: fecal egg count per gram of stool, expressed in eggs per gram of stool (EPG); SD: standard deviation; BMIAZ: body mass index for age z-score; HAZ: height-for-age z-score. The* p*-values in bold indicate a significant difference between both arms

Scores of the school WASH questionnaires are summarized in Table 2 and those of the student WASH-KAP questionnaire in Table 3. The original responses to these questionnaires are summarized in Additional file 3 and Additional file 4, respectively. At baseline, water was always available in ten schools (41.7%), available only in the rainy season in four schools (16.6%) and had to be brought to the school at all times from somewhere else in the remaining ten schools (41.7%). Only six schools (25.0%) treated water with chlorine or boiled it before use. Ten schools did not provide an allotted time period to wash hands before lunch (41.7%). In 15 schools (62.5%), there was no ‘School WASH’ club. Almost all schools had pit latrines with a cement slab (*n* = 22, 91.7%) and with good roof conditions (*n* = 23, 95.8%). However, most of the latrines were reportedly unclean/very unclean (*n* = 21, 95.8%). In most cases, the latrines were cleaned by students (*n* = 22, 91.7%) and were cleaned less than twice a week (*n* = 21, 91.7%). Open defecation practices were observed in 14 school compounds (*n* = 58.3%).

**Table 2:** Baseline response of the 24 schools to 17 questions about water, sanitation and hygiene status of the schools

Question/variable	Control (*n* = 12)	Intervention (*n* = 12)	*p*-value for difference between arms
Any kind of water source availability at school	0.5833	0.5833	1.000
Any kind of water treatment before drinking	0.0833	0.4167	0.071
School WASH club presence at school	0.4167	0.3333	0.705
≥ 1000 l water container in school	0.5833	0.75	0.417
Designated time slot in school day for hand washing	0.5833	0.5833	1.000
Latrine types (pit/ventilated/ improved pit latrine)	1	0.9167	0.359
Latrine for school children with disabilities	0.0833	0	0.359
Latrine cleaned by hired cleaners	0.0833	0.0833	1.000
Latrine cleaning frequency (daily/twice weekly)	0.0833	0.1667	0.580
Latrine floor in good condition	1	0.75	0.078
Latrine wall with better privacy	0.9167	0.9167	1.000
Latrine roof in good condition	0.9167	0.9167	0.359
Latrine hole covering availability	0	0	N/A
Latrine floor has good cleanliness	0.1667	0.0833	0.580
Latrine wall has good cleanliness	0.5	0.6667	0.437
Lack of fly infestation in toilets	0.25	0.0833	0.304
Lack of open defecation observed in school compound	0.3333	0.5000	0.437
Total WASH score	7.583	7.833	0.661

**Table 3:** Baseline knowledge, attitudes and practices of school children towards water, sanitation and hygiene

Question/Variable	Control arm (*n* = 122)	Intervention arm (*n* = 86)	*p*-value for difference between arms
Do you always wash your hands after defecation?	0.5082	0.5581	0.479
Do you always wash your hands after urination?	0.1393	0.3605	** < 0.001**
Do you always wash your hands before eating?	0.8525	0.9419	**0.044**
Do you always wash your hands when they are dirty?	0.7377	0.7907	0.380
Do you mostly wash your hands with soap or ash?	0.5328	0.3721	**0.023**
When you have to urinate at school, do you always use the toilet?	0.7541	0.7674	0.826
When you have to defecate at school, do you always use the toilet?	0.9754	0.9535	0.391
When you have to urinate at home, do you always use the toilet?	0.6148	0.5814	0.630
When you have to defecate at home, do you always use the toilet?	0.9344	0.8953	0.313
In your opinion is there a problem with the toilets at school?	0.3525	0.4884	0.050
In your opinion is there a problem with the toilets at home?	0.3115	0.2907	0.750
Do you ever miss school because of the toilets being unpleasant or not private enough?	0.9672	1	0.092
Do you ever carry water to school?	0.4672	0.4767	0.894
Are you ever thirsty at school and do not have water?	0.2295	0.3488	0.060
Total WASH-KAP score	8.377	8.826	0.1315

The* p*-values in bold indicate a significant difference between arms

The student WASH-KAP showed that 110 school children (52.9%) washed their hands after defecation and that 185 (88.9%) washed their hands before eating. A large majority of the school children always used the latrine (*n* = 201, 96.6%) at school and the latrine at home (*n* = 191, 91.8%). One hundred twenty-three (58.3%) and 145 (70.0%) of the school children reported that they did not have problems using the latrine in school or in their homes, respectively. Among the 110 (52.9%) school children who carry water to school, 45 (41.3%) get water from different sources, such as rivers and lakes. One hundred fifty (72.1%) school children reported that there had been times when they were thirsty and did not get water to drink while at school, and 35 (17.9%) of the school children went to a river once every week for recreation or swimming.

### Implementation of interventions

**Fig. 3:**
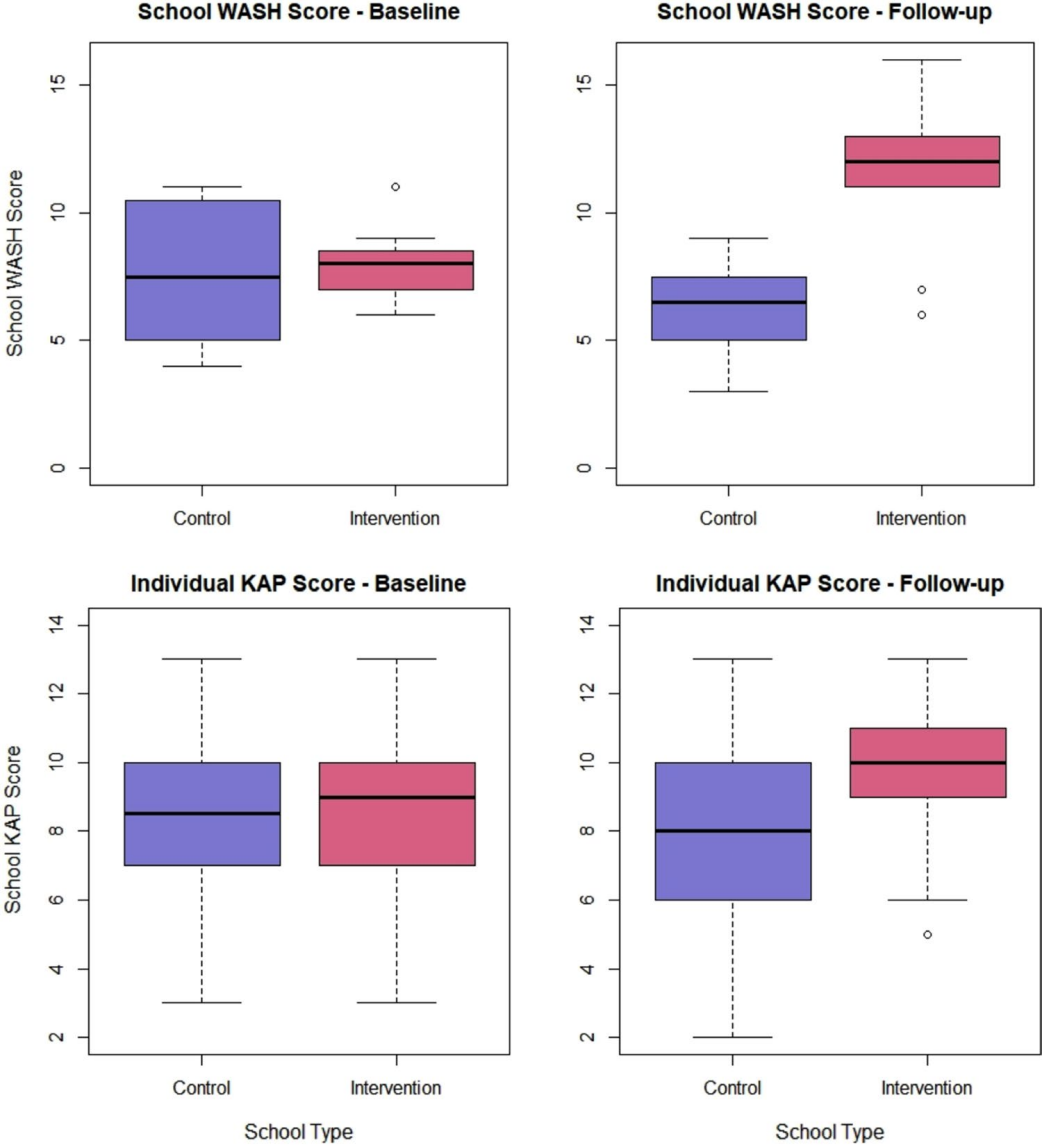
Change in total score of school WASH and student WASH-KAP in the intervention arm. These box plots represent the total score of the school WASH (top panel) and student/individual WASH-KAP (below panel) at baseline (2013) and follow-up (2015). The gray areas represent the 95% confidence intervals

Figure 3 illustrates the impact of the interventions on the total score of the school WASH and student WASH-KAP questionnaires in the intervention arm between 2013 and 2015. For both questionnaires, there was a significant increase in total score over time, indicating that the intervention was successfully implemented. For school WASH, the mean (SD) total score significantly increased from 7.83 (± 1.4) in 2013 to 11.58 (± 2.81) in 2015 (*p* = 0.012). For the student WASH-KAP, the mean total score (± SD) increased from 8.82 (± 2.19) to 9.63 (± 1.80) in 2015 (*p* = 0.031).

In Fig. 3, we further explored any spillover effect from the intervention arm to the control arm. As illustrated in this figure and confirmed by the output of the linear mixed model, the change in total score over time was significantly different between the intervention and the control arms. Indeed, the coefficient of the interaction between time and arms was significantly different from zero and positive [school WASH (± standard error (SE): 5.08 ± 1.28, *p* = 0.002); student WASH KAP (+ /SE): 1.23 ± 0.44, *p* = 0.0058], indicating that the increase in scores is steeper in the intervention arm, hence suggesting no to little spillover of the interventions. The models also indicated no significant difference in score between arms at baseline [school WASH (+/SE): 0.25 ± 0.90, *p* = 0.78); student WASH KAP (+/SE): 0.44 ± 0.31, *p* = 0.15)] and a significant main effect of time [school WASH (+/SE): − 1.33 ± 0.90, *p* = 0.15); student WASH KAP (+/SE): − 0.41 ± 0.28, *p* = 0.15)].

**Fig. 4:**
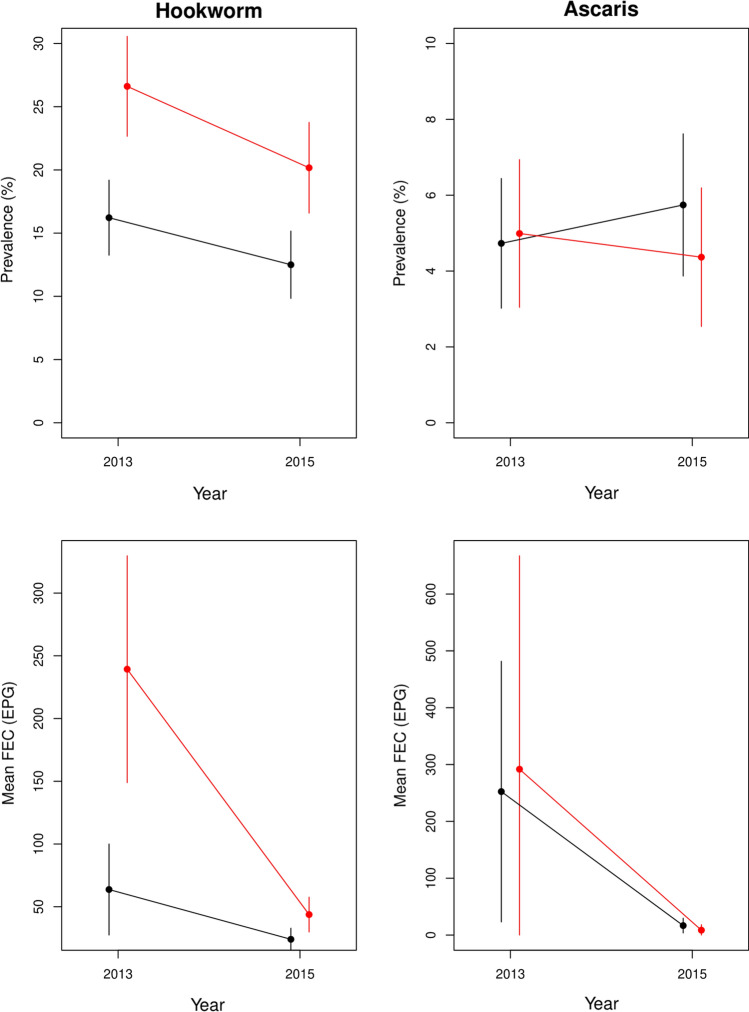
Prevalence of any soil-transmitted helminth infection over time for both arms. The line plots represent the change in prevalence in any soil-transmitted helminth infection over time for the control and intervention arms. The gray areas represent the 95% confidence intervals

### Effect of interventions on health outcomes

#### Prevalence and intensity of infection

Table 4 summarizes the outcome of the binomial generalized linear mixed model for any STH infections. Although the value of the coefficient of the interaction between the arms and time (in years) was negative, suggesting that the intervention resulted in an additional reduction in STH infections, this effect was not significant [coefficient of the interaction (± SE) = − 0.04 ± 0.11, *p* = 0.689]. Other factors that were significantly associated with a change in infection were time, school WASH score at baseline and sex. Over time, the occurrence of any STH infections significantly decreased, and, as illustrated in Fig. 4, most of the reduction occurred in the first year of the study. Thereafter, the effect plateaued. The total score on the school WASH at baseline was significantly associated with the infections, children from schools with a higher score less likely to be infected [coefficient (± SE) = − 0.21 ± 0.10, *p* = 0.048]. Finally, girls were significantly less likely to be infected than boys [coefficient (± SE) = − 0.24 ± 0.09, *p* = 0.01].

**Table 4:** The output of the linear mixed model for any soil-transmitted helminth infections and nutritional indicators

Variables	Any STH	HAZ	BMIAZ	Hemoglobin concentrations
		Estimate (SE)	Est (SE)	Est (SE)
Age at baseline (in years)	0.01 (0.02)	**0.20 (0.01)**	− **0.03(0.01)**	**0.13 (0.01)**
Time (in years)	− **0.17 (0.07)**	− 0.02 (0.10)	0.11 (0.07)	**0.39 (0.04)**
Intervention arm (vs. control arm)	0.20 (0.45)	− 0.41 (0.44)	0.47(0.22)	0.20 (0.31)
Female (vs. male)	− **0.24 (0.09)**	− 0.11 (0.06)	**0.41 (0.05)**	− **0.26 (0.07)**
WASH score at baseline	− **0.21 (0.10)**	0.09 (0.09)	− 0.05 (0.04)	− 0.006 (0.07)
Change wash score	NA	0.07 (0.06)	− 0.04 (0.02)	− 0.04 (0.03)
Intervention arm * time (in years) (vs. control arm * time)	− 0.04 (0.11)	0.12 (0.14)	− 0.06(0.10)	− **0.16 (0.06)**

The analyzed dataset contained 1046 children from 24 schools from 2013, 2014 and 2015. Values in bold indicate a significant difference between arms (*p* < 0.05)

#### Nutritional status indicators

**Fig. 5:**
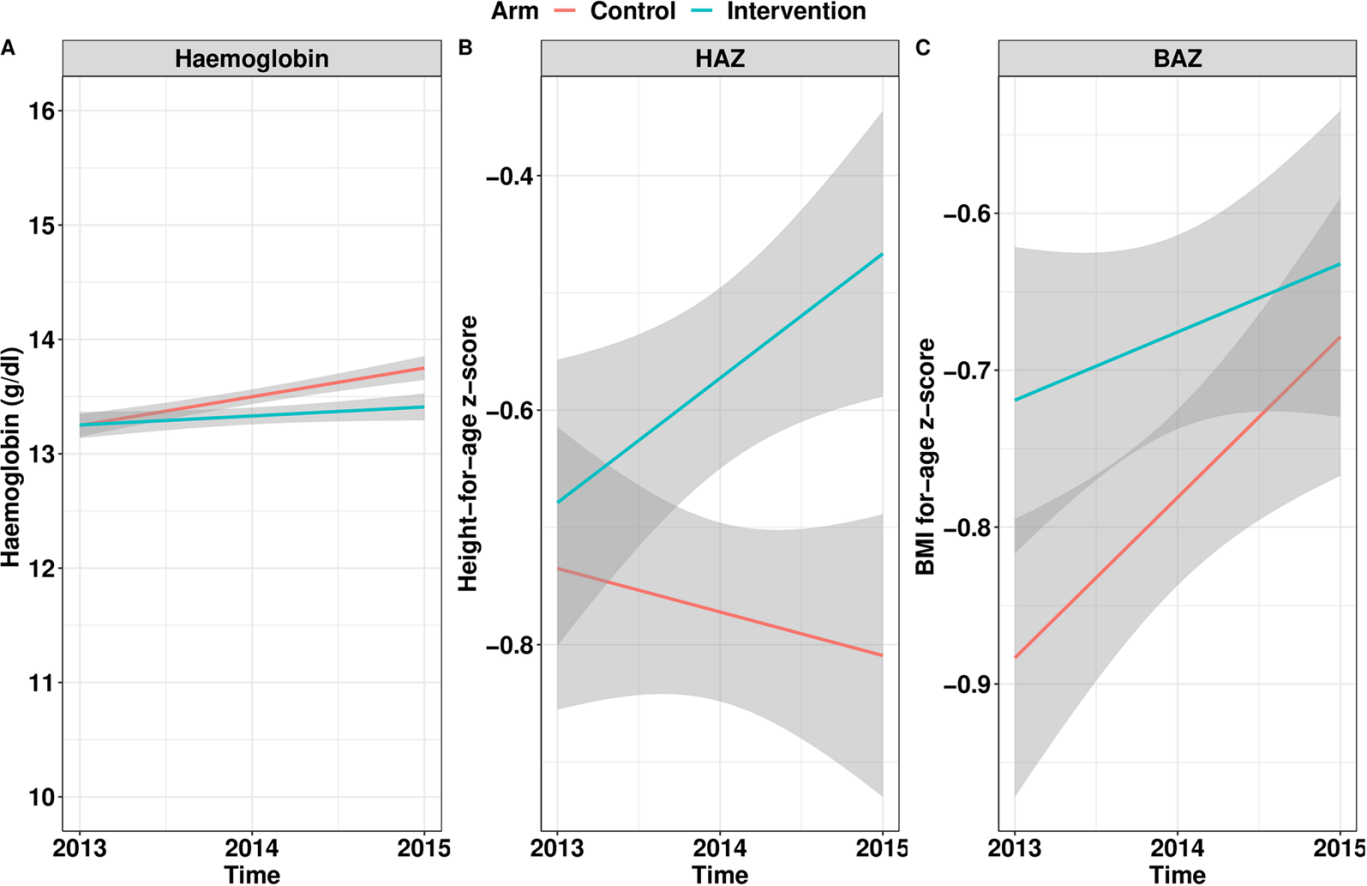
Change in nutritional status indicators for both arms. The line plots represent the change in hemoglobin concentration (Hb; in g/dl); Panel **A**), height-for-age z-score (HAZ; Panel **B**) and BMI-(body mass index)-for-age z-score (BMIAZ; Panel **C**) across schools in the control (black lines) and intervention arm (red lines). The gray areas represent the 95% confidence intervals

This analysis included the 1073 students for whom longitudinal demographic, parasitological and Hb concentration and anthropometrics were available at 2013, 2014 and 2015. Table 4 summarizes the outcome of the mixed models for each of the three nutritional status indicators (HAZ, BMIAZ and Hb concentration). The models indicated that none of the coefficients of the interactions between the arms and time were significantly different for any nutritional indicator (HAZ: 0.12 ± 0.14, *p* = 0.4007; BMIAZ: − 0.06 ± 0.10, *p* = 0.5265), except for Hb concentration. For this indicator a negative coefficient significantly different from zero was observed (− 0.16 ± 0.06, *p* = 0.0060), suggesting that the change in Hb concentration over time was lower for children in the intervention arm compared to those in the control arm (Fig. 5).

Other factors that were significantly associated with a change in nutritional indicators were age, sex and time. The coefficient of age was significantly different from zero for each of the nutritional indicators but the sign differed across indicators. While the HAZ and Hb concentrations increased as a function of age at baseline, BMIZs were lower for older children. A significant impact of sex was observed for BMIZ and Hb concentration. While BMIZs were higher for girls than boys (0.41 ± 0.05, *p* = 0.0001), the Hb concentration was lower in girls (− 0.26 ± 0.07, *p* = 0.0002). A significant change over time was observed for Hb concentration only, the Hb concentration increasing over time (0.39 ± 0.04, *p* = 0.0001).

#### Comparison of school children who completed the study and those who were lost to follow-up

When comparing those students followed up with those lost to follow-up, we summarized the comparisons for nutritional indicators and any STH infections in Table 5. At baseline, those in the intervention arm had a higher level of STH prevalence than those in the control arm (z = − 3.334, *p* < 0.001), contributed by a higher level of hookworm prevalence (z = − 4.165, *p* < 0.001). Students in the intervention arm also had a higher prevalence of severe anemia (z = − 2.133, *p* = 0.033) and a lower BMIAZ (t = − 2.350, df = 976.9, *p* = 0.019).

**Table 5:** Comparison of school children who completed the study and those who were lost to follow-up

Variables	Overall (*n* = 3698)	Lost to follow-up (*n* = 2625)	Followed up (*n* = 1073)	*p*-value
Male, *n* (%)	1941 (52.48)	1332 (50.74)	609 (56.75)	**0.0010**
Mean age (SD)	11.8 (2.09)	11.9 (2.16)	11.7 (1.91)	**0.0157**
Median weight (interquartile range, IQR)	33.4 (13)	33.7 (13.9)	33 (11.3)	0.0634
Median height (IQR)	144 (16)	144 (17)	144 (14.4)	0.5716
HAZ, median (IQR)	− 0.75 (1.97)	− 0.76 (2.03)	− 0.67 (1.93)	0.0875
BMIAZ, median (IQR)	− 0.61 (1.48)	− 0.55 (1.50)	− 0.73 (1.43)	**0.0003**
Hemoglobin concentration, mean (SD)	13.3 (1.64)	13.2 (1.68)	13.3 (1.54)	0.7394
Any STH infections, *n* (%)	858 (23.20)	583 (22.20)	275 (25.62)	**0.0283**

The* p*-values in bold indicate a significant difference between both arms

## Discussion

Large-scale deworming programs have been the main tool to control STH and SCH infections for many years [29], and data now also confirm that they have been successful in reducing infection levels and hence related morbidity [30, 31]. It has long been postulated that to further suppress the infection to low levels that may result in a break in transmission, complementary interventions such as WASH improvements and health education are required [32, 33]. However, the evidence for the ancillary benefits of these interventions is not robust [34, 35]. We therefore assessed the additional impact of a deworming program combined with a food program, improved WASH infrastructure and health education at school on the prevalence and intensity of STH and SCH and selected nutritional indicators in school children.

### The intervention resulted in an increased school WASH score and individual KAPs

Our WASH data indicated that the school WASH intervention was successfully implemented as planned by the project. This is evidenced by the overall increase in school WASH scores, including an increase in latrine cleanliness, having a dedicated cleaner, having latrines available for students with disabilities and a decrease in the need to treat water before drinking. At this level, the intervention also improved students’ KAP towards WASH to prevent STH and SCH infections, including increases in hand-washing after defecation and urination and an improvement in the perceived quality of the school latrine. However, in the control arm the school WASH deteriorated over time, providing evidence for no spillover effect of the intervention on the control communities. However, the student KAP showed some improvements, which might be linked with the repeated administration of the questionnaire.

### Poor evidence of an additional impact of improved WASH infrastructure and health education

Despite the successful implementation of the project, the intervention did not result in reductions in helminth infections or infection-related morbidity that were significantly greater than those observed in the control group (which received deworming and lunch only). While some other studies have reported that improvements in WASH and increased access to clean water led to reduced STH and SCH infections [35–37], and that the additional impact of WASH on the prevalence of STH infections was observed when combined with other interventions, such as health education and regular deworming [18, 19, 38–40], those findings were not replicated here. These results, however, are consistent with several other studies conducted in children < 5 years of age, which assessed the sole or combined effect of WASH with nutritional supplements and deworming and showed no additional effect of household-level implemented WASH and a modest effect of nutritional supplements on child growth [11, 41]. Few studies have been conducted on school children, and, as Grimes et al. [35] reported in their meta-analysis, the quality of available studies is generally poor and observational in nature [35]. The ongoing randomized controlled trial conducted in schools in Manila (the Philippines), which is assessing the impacts of school-based WASH interventions on school children's health literacy, handwashing and nutrition status, is a good example of a strong design that may provide robust evidence [42].

Potential explanations for this weak impact of improved WASH intervention on both helminth infection and nutritional indicators in our study are multifactorial. First, if occurring, the additional impact of the intervention might be small and therefore require a larger sample size. Indeed, the largest impact on helminth infections is likely due to the deworming program itself, as evidenced by the uniform drop in infections across both arms (Fig. 2). Second, it is possible that a longer follow-up period (only 2 years of follow-up in this study) would result in more significant differences in both helminth infections and nutrition-related indicators. Third, the relatively low prevalence of helminth infections, stunting and thinness observed in this study may have resulted in a very small effect size of the intervention, if not null. Fourth, the effect of improved dietary intake through the food program might have a larger effect on the nutritional status of school children. Moreover, dietary intake and morbidity other than STH and SCH such as diarrhea and malaria, which are key factors in nutritional outcomes, especially in younger children, were not assessed. Finally, the interventions—both WASH and behavior change—were focused on schools only. Improved WASH facilities and hygiene education at the schools do not necessarily reflect changes in the community where fecal contaminations might occur through low coverage of sanitation infrastructure and hand contamination when hands are washed with water only and during food preparations [14]. 

We also recognize some study limitations due to the nature of the study design, qualitative data and the high dropout rate. We applied a quasi-random design with a limited ability to have comparable control and intervention groups at baseline and consequently to demonstrate a causal effect of the intervention and the health outcomes. The study assessed the WASH at schools and the student-KAP toward WASH based exclusively on reports which are subjective and could be biased. Finally, the study experienced high dropout between baseline and follow-up, particularly in girls and among participants who are older and thinner and have lower prevalence of any STH infection and hookworm infection. Note that the impact of the intervention was associated with being female (on any STH, BMIAZ and Hb concentrations) and age (on HAZ, BMIAZ and Hb concentrations). It is possible that the selective dropout pattern might have diminished the potential effect of this intervention.

## Conclusions

From the perspective of the project implementation, the interventions were successful in improving WASH scores at both the school and individual level. These improvements persisted after 2 years of follow-up. Although there were significant decreases in STH infections in both arms, there was not a statistically significant increased impact of the intervention on helminth infection, except for on hookworm infections, or on nutritional indicators. We recommend comprehensive and rigorous studies that can quantitatively assess the impact of WASH interventions at the level of both schools and communities. As efforts are made to improve the coverage of WASH interventions at both schools and communities, we emphasize that the implementation should be robustly designed and implemented to achieve the goals of reducing exposure to pathogens and delivering health gains.

### Supplementary Information

**Additional file 1: Table S1.** The demographic characteristics, status of helminth infections and nutritional status at baseline compared between age categories.

**Additional file 2: Table S2.** The baseline response of the 24 schools to 17 questions about water, sanitation and hygiene.

**Additional file 3: Table S3.** The baseline response of 208 students to 17 questions to assess their KAP towards WASH.

## Data Availability

The data supporting the finding will be deposited in a publicly available database.

## Abbreviations

BMIAZ: Body mass index-for-age z-score
DHS: Demographic and Health Survey
FAO: Food and Agriculture Organization
HAZ: Height-for-age z-score
Hb: Hemoglobin
KAP: Knowledge, attitudes and practices
STH: Soil-transmitted helminths
SCH: Schistosomes
SD: Standard deviation
SNNPR: Southern Nation, Nationalities Peoples Region
SNV: Stichting Nederlandse Vrijwilligers’
UN-WFP: United Nations World Food Program
WASH: Water, sanitation and hygiene
WHO: World Health Organization
