# Supplementary material for: Investigating the effect of a school-based WASH intervention on soil-transmitted helminth and schistosome infections and nutritional status of school children in Ethiopia: a quasi-experimental study
Source: Parasit Vectors. 2024 Mar 14;17:130. doi: 10.1186/s13071-024-06155-2 (PMC10938701; doi:10.1186/s13071-024-06155-2)
Supplement: Supplementary file 1 — Additional file 1: Table S1. The demographic characteristics, status of helminth infections and nutritional status at baseline compared between age categories. [file 13071_2024_6155_MOESM1_ESM.docx]

**S1 Table. The demographic characteristics, status of helminth infections, and nutritional status at baseline compared between age categories.** FEC: fecal egg count per gram of feces, expressed in eggs per gram of stool (EPG). M = Male. SD = standard deviation. Prev = prevalence. BMIAZ = body mass index for age z-score. HAZ = height-for-age z-score.

|  | **Metric** | **Value** | **Total (n = 1,073)** | **Age <12yrs (n = 475)** | **Age >= 12yrs (598)** | **Diff. between age categories.  *p-value*** |
| --- | --- | --- | --- | --- | --- | --- |
| ***Demography*** | |  |  |  |  |  |
|  | Sex (M, %) |  | **56.8** | 56.6% | 56.9% | 0.941 |
|  | Mean Age [SD] |  | **11.7 [1.9]** | - | - | - |
| ***Helminth infection*** | |  |  |  |  |  |
|  | Any STH | Prev. (%) | **25.6** | 26.5% | 24.9% | 0.550 |
|  | *Ascaris* | Prev. (%) | **4.8** | 4.2% | 5.4% | 0.382 |
|  |  | Mean FEC (EPG) | **270.1** | 60.18 | 436.8 | 0.052 |
|  | *Trichuris* | Prev. (%) | **0.7** | 0.6% | 0.8% | 0.695 |
|  |  | Mean FEC (EPG) | **7.0** | 0.45 | 12.16 | 0.093 |
|  | Hookworm | Prev. (%) | **20.9** | 22.1% | 19.9% | 0.380 |
|  |  | Mean FEC (EPG) | **142.4** | 150.2 | 136.3 | 0.769 |
|  | *Schistosoma mansoni* | Prev. (%) | **0.2** | 0.2% | 0.2% | 0.872 |
|  |  | Mean FEC (EPG) | **<0.1** | 0.05 | 0.04 | 0.872 |
| ***Nutritional indicators*** | |  |  |  |  |  |
|  | Haemaglobin | g/dl [SD] | **11.4 [1.5]** | 11.2 [1.4] | 11.5 [1.6] | **<0.001** |
|  | Anaemia | Severe (%) | **2.4** | 2.1% | 2.7% | 0.541 |
|  |  | Moderate (%) | **31.7** | 37.1% | 27.4% | **0.001** |
|  |  | Mild (%) | **22.9** | 15.8% | 28.6% | **<0.001** |
|  | BMIAZ | z-score [SD] | **-0.74 [1.08]** | -0.63 [1.00] | -0.82 [1.13] | **0.004** |
|  | Thinness (%) | Severe (%) | **1.7%** | 0.6% | 2.5% | **0.011** |
|  |  | Moderate (%) | **9.5%** | 6.1% | 12.2% | **<0.001** |
|  | HAZ | z-score [SD] | **-0.66 [1.51]** | -0.23 [1.58] | -1.00 [1.36] | **<0.001** |
|  | Stunting (%) | Severe (%) | **5.9%** | **3.4%** | 7.9% | **0.001** |
|  |  | Moderate (%) | **11.0%** | **8.8%** | 12.7% | **0.041** |
