# Supplementary material for: Investigating the effect of a school-based WASH intervention on soil-transmitted helminth and schistosome infections and nutritional status of school children in Ethiopia: a quasi-experimental study
Source: Parasit Vectors. 2024 Mar 14;17:130. doi: 10.1186/s13071-024-06155-2 (PMC10938701; doi:10.1186/s13071-024-06155-2)
Supplement: Supplementary file 2 — Additional file 2: Table S2. The baseline response of the 24 schools to 17 questions about water, sanitation and hygiene. [file 13071_2024_6155_MOESM2_ESM.docx]

**S2 Table. The baseline response of the 24 schools to 17 questions about water, sanitation, and hygiene.**

| **Question** | **Response** | **Total (%)**  **(n = 24)** | **Control**  **(n = 12)** | **Intervention**  **(n = 12)** |
| --- | --- | --- | --- | --- |
| Availability of a water source within the school compound | Water is always available | 10 (41.7) | 5 | 5 |
|  | Water is only available during the rainy season | 4 (16.6) | 2 | 2 |
|  | Water is always brought to the school from elsewhere | 10 (41.7) | 5 | 5 |
| Treatment of drinking water | No treatment | 18 (75.0) | 11 | 7 |
|  | Adding chlorine / wuha agar / boiling | 6 (25.0) | 1 | 5 |
| Presence of a school WASH club | Yes | 9 (37.5) | 5 | 4 |
|  | No | 15 (62.5) | 7 | 8 |
| Presence of a large (capacity at least 1,000 L) container or tank for water storage | Yes | 16 (66.7) | 9 | 7 |
|  | No | 8 (33.3) | 3 | 5 |
| Allotted time period for students to wash hands before eating | Yes | 14 (58.3) | 7 | 7 |
|  | No | 10 (41.7) | 5 | 5 |
| Type of latrine | Pit latrines with cement slab | 22 (91.7) | 11 | 11 |
|  | Pit latrines without cement slab (made of wood) | 1 (4.2) | 0 | 1 |
|  | Ventilated improved pit latrines (VIPs) | 1 (4.2) | 1 | 0 |
| Separate latrine for people with disabilities | Yes | 1 (4.2) | 1 | 0 |
|  | No | 23 (95.8) | 11 | 12 |
| Latrine cleaned by | Students | 22 (91.7) | 11 | 11 |
|  | Hired Cleaners | 0 (0.0) | 0 | 0 |
|  | Others | 2 (8.3) | 1 | 1 |
| Latrine cleaning frequency | Daily | 3 (12.5) | 1 | 2 |
|  | Twice weekly | 5 (20.8) | 3 | 2 |
|  | Weekly | 11 (45.8) | 4 | 7 |
|  | Less than weekly | 5 (20.8) | 4 | 1 |
| Latrine floor structural condition | Good, no signs of damage | 21 (87.5) | 12 | 9 |
|  | Cracked but in place | 3 (12.5) | 0 | 3 |
| Privacy of latrine walls | Has private walls (no holes) | 22 (91.7) | 11 | 11 |
|  | Has walls with holes | 2 (8.3) | 1 | 1 |
| Condition of the roof | No holes (completely waterproof) | 23 (95.8) | 11 | 12 |
|  | Has holes (somewhat waterproof) | 1 (4.2) | 1 | 0 |
| latrine hole covering | Present but hole is uncovered | 1 (4.2) | 0 | 1 |
|  | No hole covering present | 23 (95.8) | 12 | 11 |
| Cleanliness of latrine floors | Clean | 3 (12.5) | 2 | 1 |
|  | Unclean | 11 (45.8) | 6 | 5 |
|  | Very unclean | 10 (41.7) | 4 | 6 |
| Cleanliness of latrine walls | Clean | 14 (58.3) | 6 | 8 |
|  | Unclean | 8 (33.3) | 5 | 3 |
|  | Very unclean | 2 (8.3) | 1 | 1 |
| Presence of flies or other insects | No | 4 (17.4) | 3 | 1 |
|  | Some (less than about 20) | 12 (52.4) | 6 | 6 |
|  | Many (more than about 20) | 7 (30.4) | 3 | 4 |
| Evidence of open defecation in the school | Yes | 14 (58.3) | 8 | 6 |
|  | No | 10 (41.7) | 4 | 6 |
