# Supplementary material for: Investigating the effect of a school-based WASH intervention on soil-transmitted helminth and schistosome infections and nutritional status of school children in Ethiopia: a quasi-experimental study
Source: Parasit Vectors. 2024 Mar 14;17:130. doi: 10.1186/s13071-024-06155-2 (PMC10938701; doi:10.1186/s13071-024-06155-2)
Supplement: Supplementary file 3 — Additional file 3: Table S3. The baseline response of 208 students to 17 questions to assess their KAP towards WASH. [file 13071_2024_6155_MOESM3_ESM.docx]

**S3 Table. The baseline response of 208 students to 17 questions to assess their KAP towards WASH.**

| **Question** | **Response** | **Total (%)**  **(n = 208)** | **Control (n = 122)** | **Intervention (n = 86)** |
| --- | --- | --- | --- | --- |
| Hand washing after defecation | Yes | 110 (52.9) | 62 | 48 |
|  | No | 98 (47.1) | 60 | 38 |
| Hand washing after urination | Yes | 48 (23.1) | 17 | 31 |
|  | No | 160 (76.9) | 105 | 55 |
| Hand washing before eating | Yes | 185 (88.9) | 104 | 81 |
|  | No | 23 (11.1) | 18 | 5 |
| Hand washing when dirty | Yes | 158 (76.0) | 90 | 68 |
|  | No | 50 (24.0) | 32 | 18 |
| Hand washing mostly by | Water only | 111 (53.4) | 57 | 54 |
|  | Soap and water | 94 (45.2) | 65 | 29 |
|  | Ash and water | 3 (1.4) | 0 | 3 |
| To urinate I always use the latrine at school | Yes | 158 (76.0) | 92 | 66 |
|  | No | 50 (24.0) | 30 | 20 |
| To defecate I always use the latrine at school | Yes | 201 (96.6) | 119 | 82 |
|  | No | 7 (3.4) | 3 | 4 |
| To urinate I always use the latrine at home | Yes | 125 (60.1) | 75 | 50 |
|  | No | 83 (39.9) | 47 | 36 |
| To defecate I always use the latrine at home | Yes | 191 (91.8) | 114 | 77 |
|  | No | 17 (8.2) | 8 | 9 |
| In your opinion is there a problem with the latrine at school | No | 123 (58.3) | 79 | 44 |
|  | Yes | 85 (40.9) | 43 | 42 |
| In your opinion is there a problem with the latrine at home | No | 145 (70.0) | 84 | 61 |
|  | Yes | 62 (30.0) | 38 | 24 |
| Do you ever miss school because of the toilets being unpleasant | Yes | 4 (1.9) | 4 | 0 |
|  | No | 204 (98.1) | 118 | 86 |
| Do you ever carry water to school? | Yes | 110 (52.9) | 65 | 45 |
|  | No | 98 (47.1) | 57 | 41 |
| Where does this water come from? | Public tap | 64 (58.7) | 49 | 15 |
|  | Other sources | 45 (41.3) | 16 | 29 |
| Are you ever thirsty at school and don't have a drink? | Yes | 150 (72.1) | 94 | 56 |
|  | No | 58 (27.9) | 28 | 30 |
| Frequency of going into the river for swimming for fun | At least once a week | 35 (17.9) | 19 | 16 |
|  | Less than once per week | 160 (82.1) | 92 | 68 |
